# Supplementary material for: A multi-functional role for the MCM8/9 helicase complex in maintaining fork integrity during replication stress
Source: Nat Commun. 2022 Aug 30;13:5090. doi: 10.1038/s41467-022-32583-8 (PMC9427862; doi:10.1038/s41467-022-32583-8)
Supplement: Supplementary file 1 — Supplementary Information [file 41467_2022_32583_MOESM1_ESM.pdf]

Supplemental Information

A multi-functional role for the MCM8/9 helicase complex in maintaining fork integrity during replication stress

Wezley C. Griffin, David R. McKinzey, Kathleen N. Klinzing, Rithvik Baratam, Achini Eliyapura, and Michael A. Trakselis

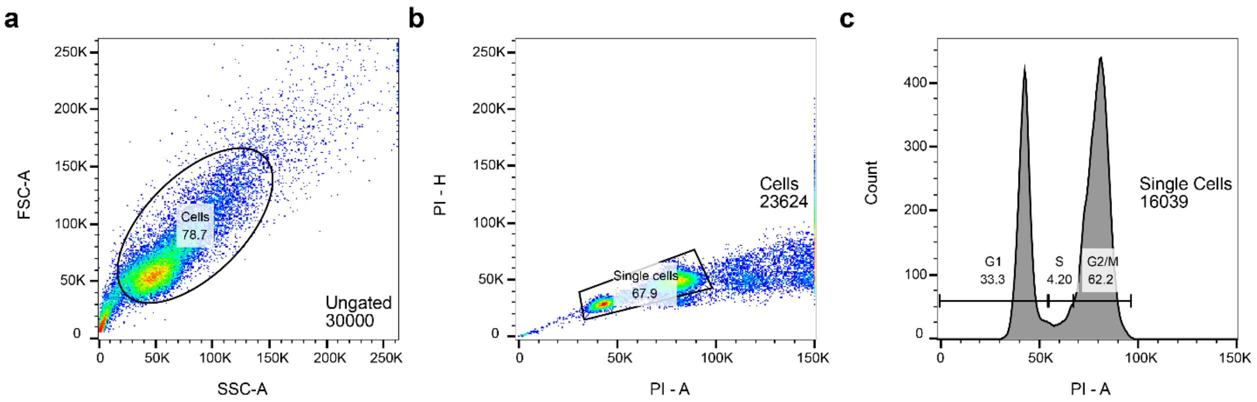

Supplemental Figure 1. FACS gating strategy for cell synchronization.
a Cells from **Figure 1c** were selected using forward scatter area (FSC-A) versus side scatter area (SSC-A) and then **b** selected for single cells using propidium iodide height (PI – H) and area (PI – A). **c** Shows the population of cells by PI - A and the gating for G1, S, and G2/M phases. Example data is for the parental 293T double thymidine block synchronization after 8-hour release.

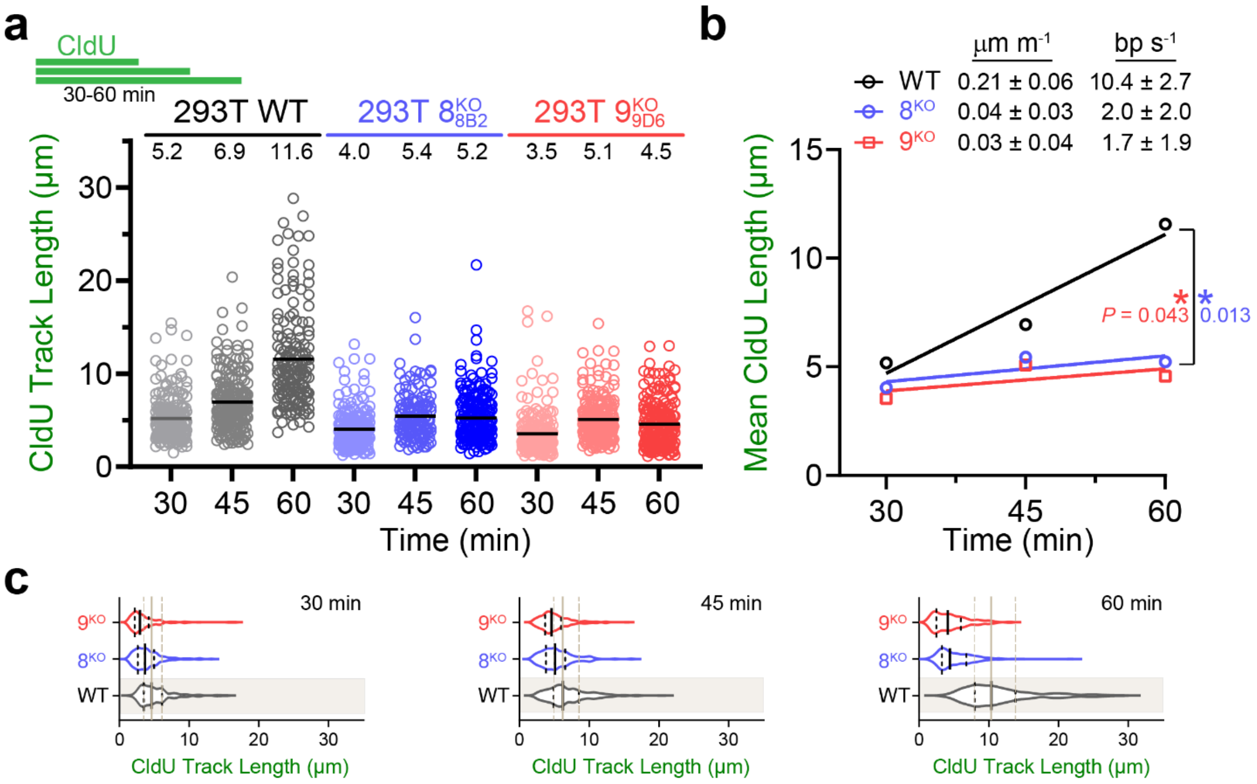

Supplemental Figure 2. Overall DNA synthesis rates are reduced in MCM8^{KO} or MCM9^{KO} clones 8B2 and 9D6.

a DNA fiber assay used to monitor the overall DNA synthesis rate. Cells were treated with 50 μM CldU for the indicated time intervals, and the CldU track lengths (>150 fibers each) were measured as a readout of replication progression for 293T WT (grey circles, ○), 8^{KO} (8B2 clone, blue circles, ○), 9^{KO} (9D6 clone, red circles, ○) cells. CldU lengths were measured with ImageJ software, and the corresponding mean value of each time point are listed above each plot and indicated with a black bar. **b** Mean CldU track length values were plotted as a function of time to obtain apparent DNA synthesis rates. A two-sided *t* test was used to calculate *P*-values between the slopes for either WT and 8^{KO} or 9^{KO} (*P< 0.05). **c** Violin plots comparing the CldU track lengths at each time point to the WT values (beige box and lines) with mean (solid line) and quartiles (dashed lines) indicated.

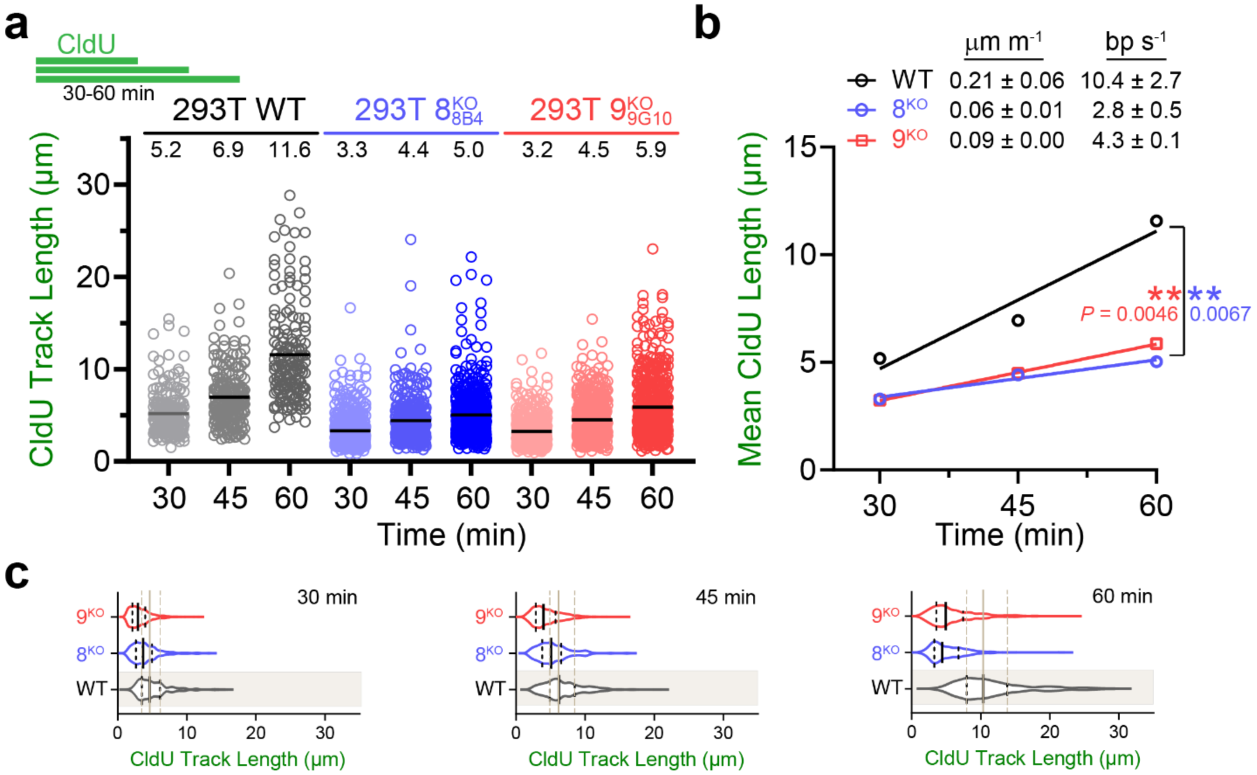

Supplemental Figure 3. Overall DNA synthesis rates are reduced in second MCM8^{KO} or MCM9^{KO} clones 8B4 and 9G10.

a DNA fiber assay used to monitor the overall DNA synthesis rate. Cells were treated with 50 µM CldU for the indicated time intervals, and the CldU track lengths (>150 fibers each) were measured as a readout of replication progression for 293T WT (grey circles, ○), 8^{KO} (8B4 clone, blue circles, ○), 9^{KO} (9G10 clone, red circles, ○) cells. CldU lengths were measured with ImageJ software, and the corresponding mean value of each time point are listed above each plot and indicated with a black bar. **b** Mean CldU track length values were plotted as a function of time to obtain apparent DNA synthesis rates. A two-sided *t* test was used to calculate *P*-values between the slopes for either WT and 8^{KO} or 9^{KO} (*P< 0.05). **c** Violin plots comparing the CldU track lengths at each time point to the WT values (beige box and lines) with mean (solid line) and quartiles (dashed lines) indicated.

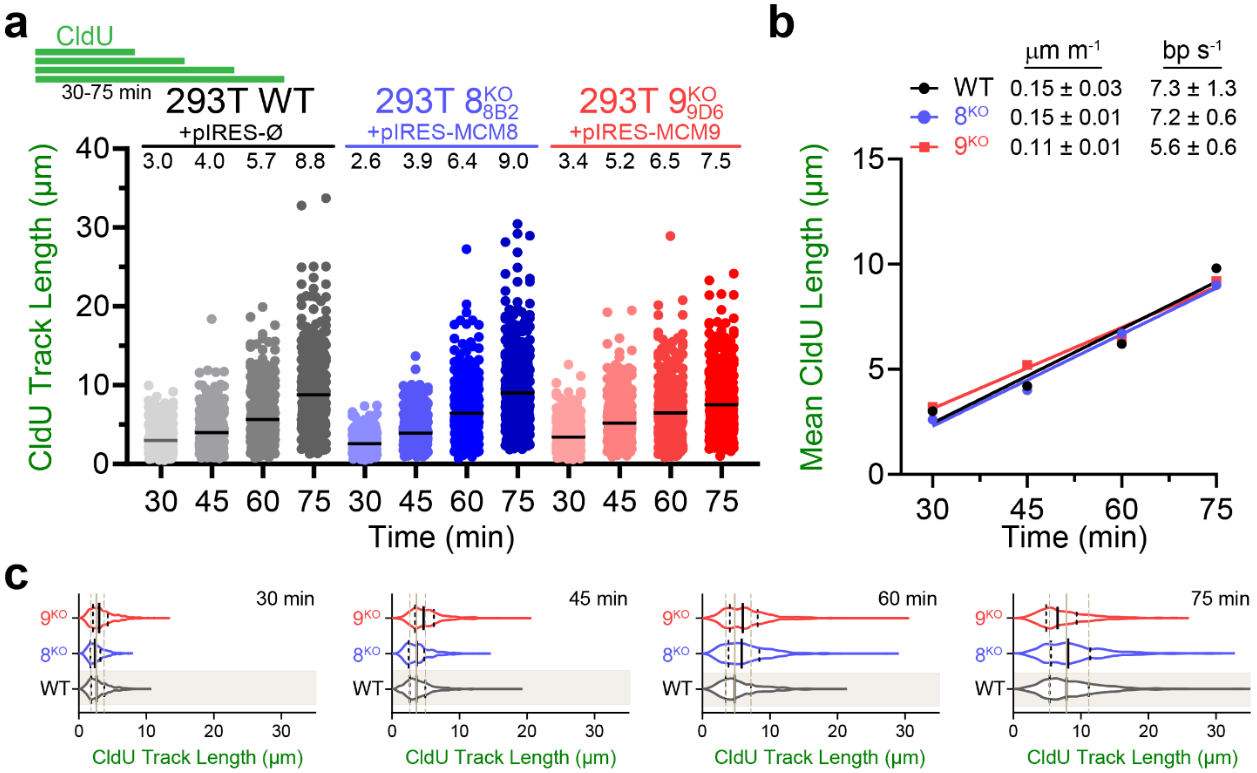

Supplemental Figure 4. Overall DNA synthesis rates are restored in when MCM8 or MCM9 is transfected back into respective knockout cells.
a DNA fiber assay used to monitor the overall DNA synthesis rate. Cells were transfected with pIRES vectors: empty (Ø) in 293T WT (grey circles, ●), MCM8 in 8^{KO} (8B2 clone, blue circles, ●), or MCM9 in 9^{KO} (9D6 clone, red circles, ●) cells prior to treatment with 50 μM CldU for the indicated time intervals. The CldU track lengths (>150 fibers each) were measured as a readout of replication progression with ImageJ software and the corresponding mean value of each time point are listed above each plot and indicated with a black bar. **b** Mean CldU track length values were plotted as a function of time to obtain apparent DNA synthesis rates. There is no significant difference in the slopes upon restoration of MCM8 or MCM9 (compare to **Supp. Figs 1** & **2**). **c** Violin plots comparing the CldU track lengths at each time point to the WT values (beige box and lines) with mean (solid line) and quartiles (dashed lines) indicated.

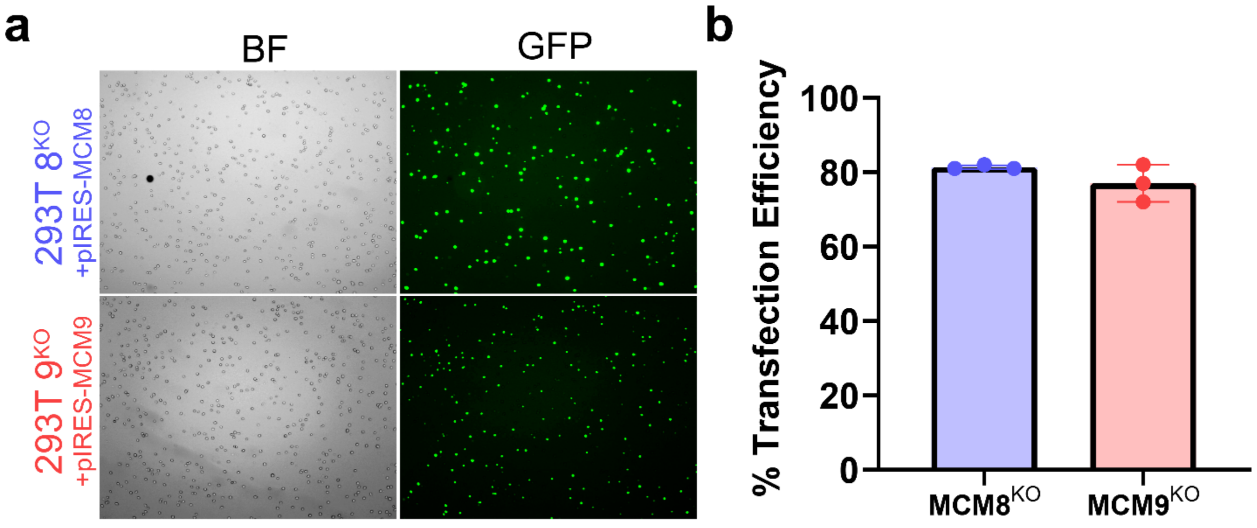

Supplemental Figure 5. Transfection efficiencies of MCM8 or MCM9 in cells.
a pIRES-MCM8 or pIRES-MCM9 plasmids were transfected into their respective knockout cell lines using TransIT-X2 (Mirus), imaged for brightfield (BF) or GFP fluorescence (*n* = 3 independent experiments) and then **b** quantified. Plots show the average and standard deviation of three independent transfection experiments for each condition.

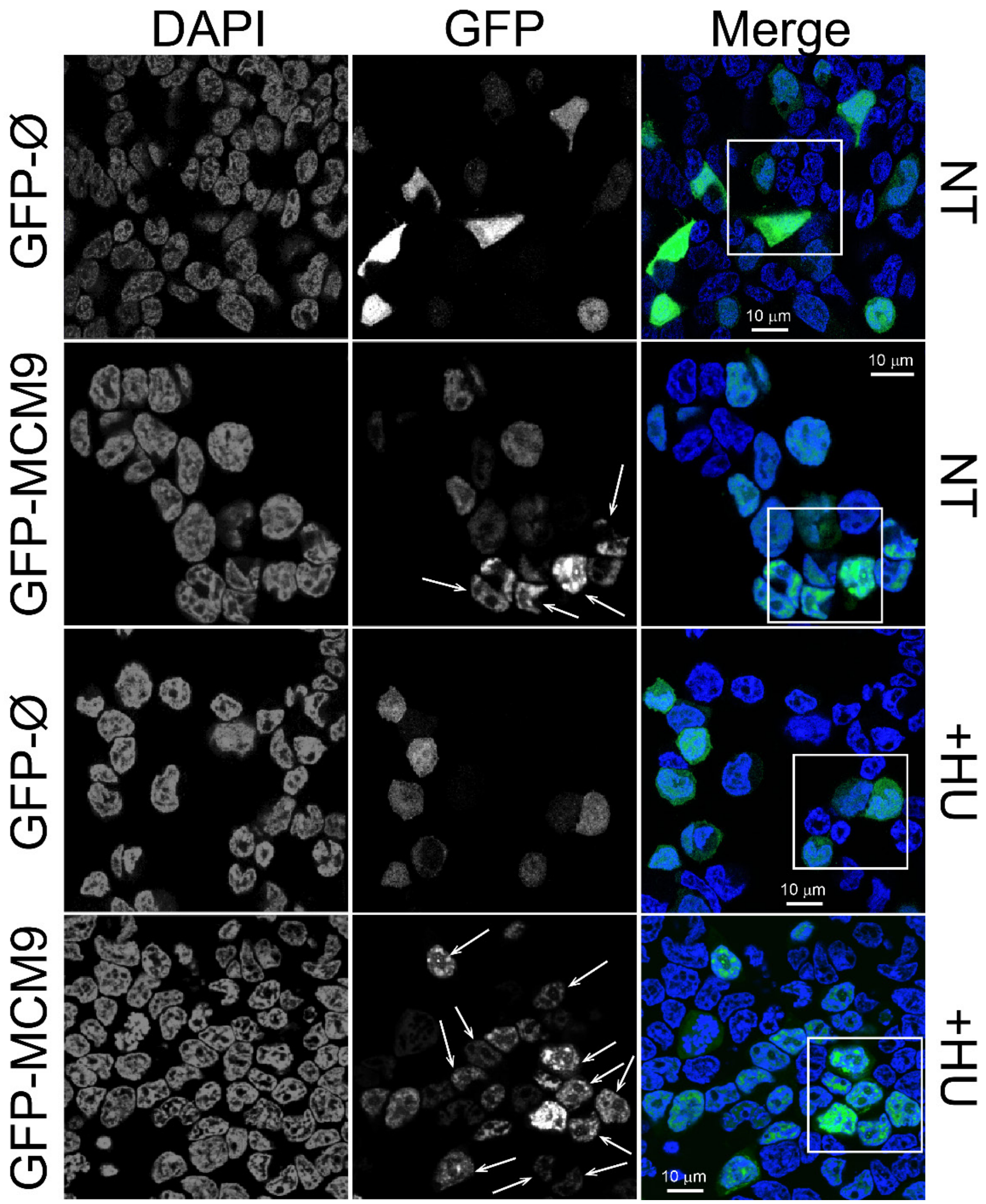

Supplemental Figure 6. MCM9 form foci in HU treated cells.
293T cells were transfected with GFP-MCM9 fusion construct or GFP alone (Ø), nontreated (NT) or treated with 2 mM HU for 4 hours (+HU), and then imaged by confocal microscopy (*n* = 2 independent experiments). White arrows indicate distinct nuclear GFP-MCM9 foci. White square boxes represent the images cropped and shown in **Figure 2a**. Scale bars represent 10 µm.

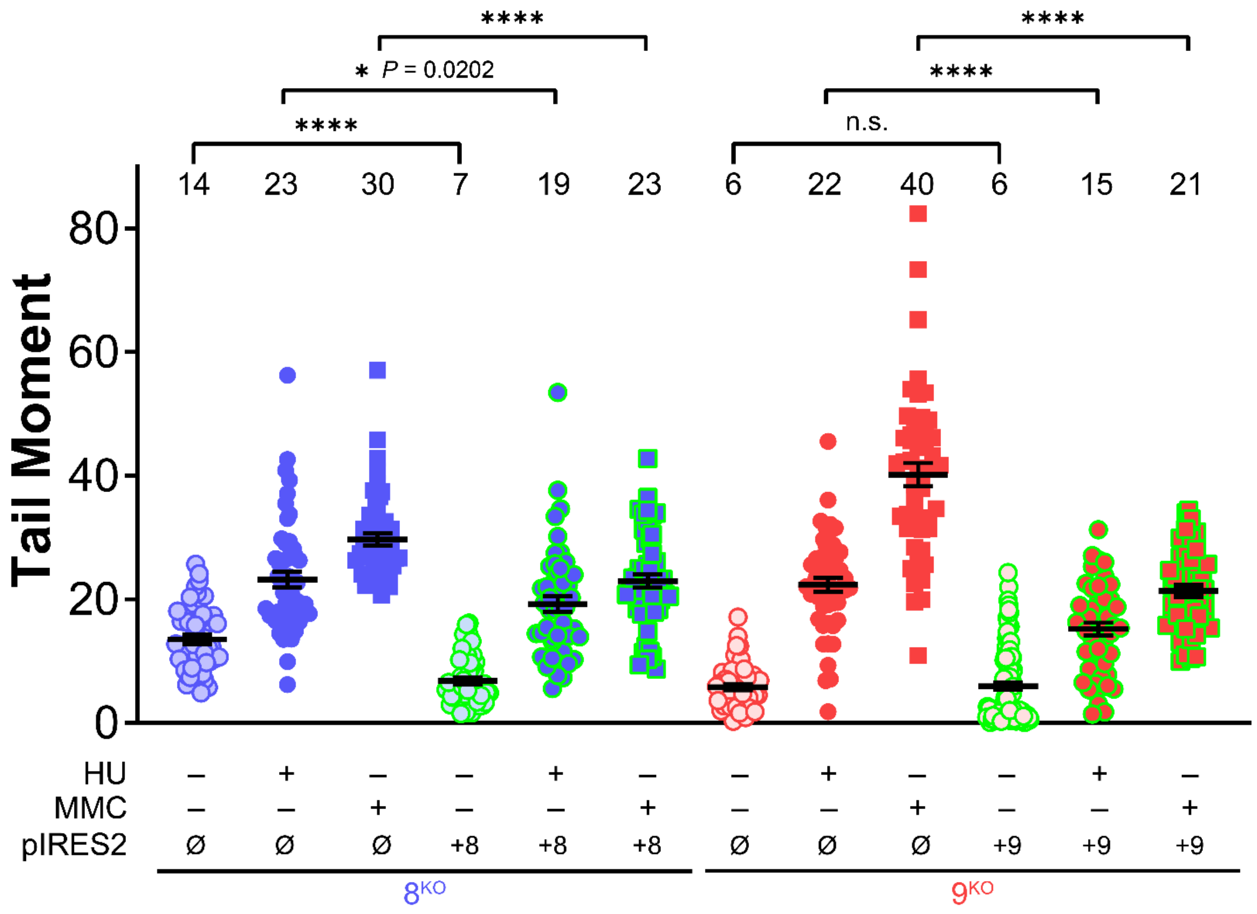

Supplemental Figure 7. Restoration of Comet tails in knockout cell lines by transfection of pIRES-MCM8 or pIRES-MCM9.

8^{KO} or 9^{KO} cells were transfected with pIRES2 empty vector (Ø), pIRES2-MCM8 (+8), or pIRES2-MCM9 (+9) (○, green outline) either nontreated (NT) (○, open circle) or treated with 2 mM HU for 4 hours (+HU) (●, closed circle) or 3 μM MMC for 6 hours (■, closed box). The comet tail moments were calculated with the mean values listed above the plots and with a black line embedded in the data and error bars represent the standard error of the mean (SEM) (n.s. nonsignificant, *P< 0.05, ***P< 0.0001, by paired two-sided t test).

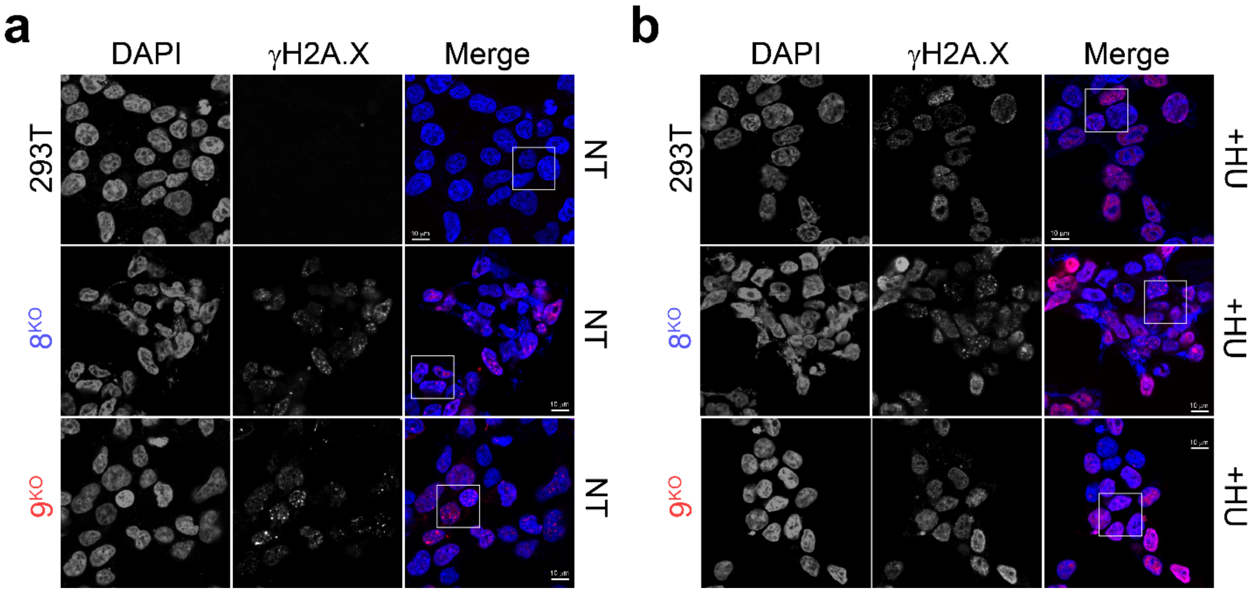

Supplemental Figure 8. Broader images of γH2A.X staining and foci in nontreated (NT) and HU treated cells. 293T, 8^{KO}, or 9^{KO} were examined for either **a** native γH2A.X foci in nontreated (NT) cells or **b** after treatment with 2 mM HU for 4 hours (+HU) and then imaged by confocal microscopy (*n* = 3 independent experiments). Scale bars represent 5 µm. White square boxes represent the images cropped and shown in **Figure 3a-b**.

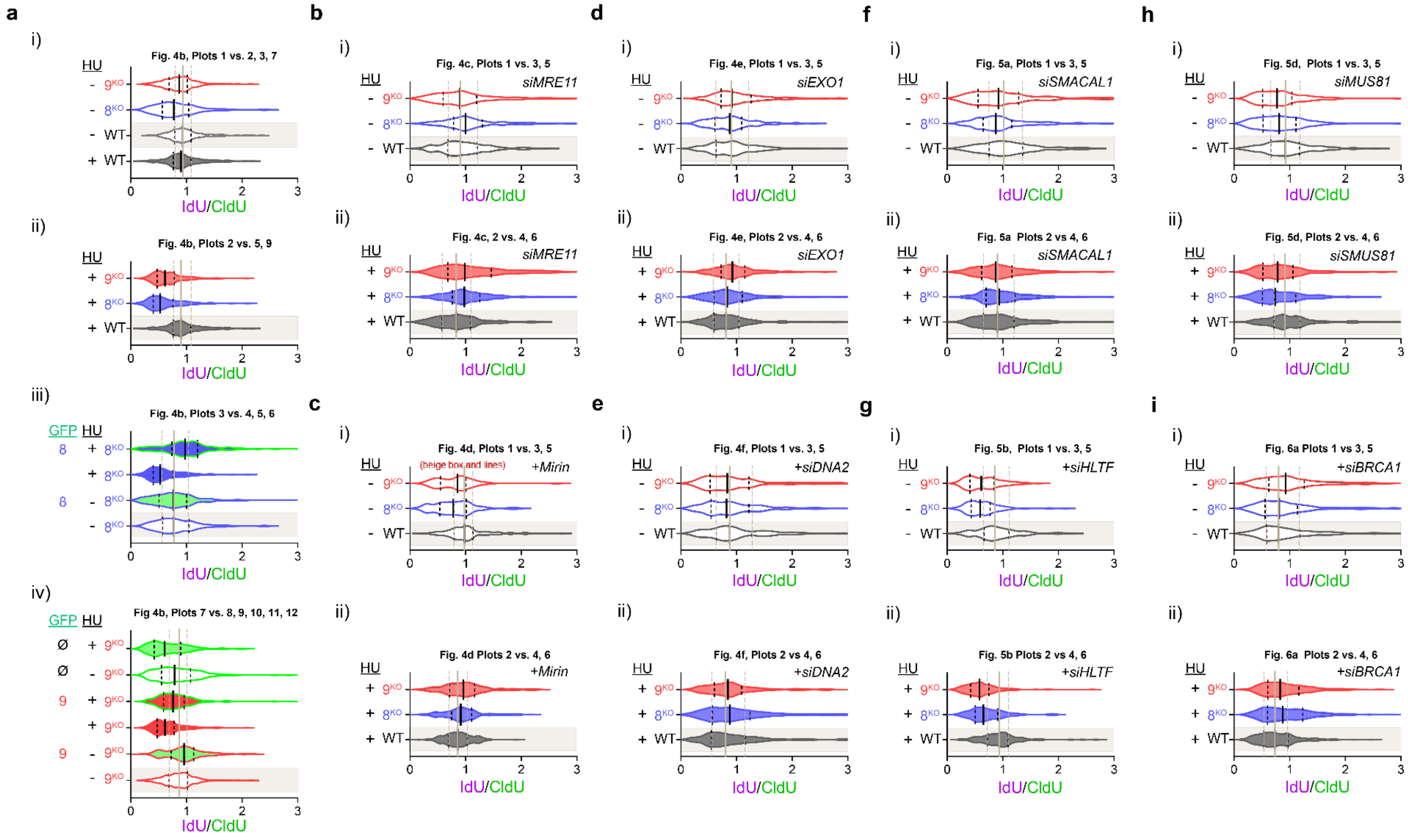

Supplemental Figure 9. Direct comparisons of quartiles for Idu/CldU ratios.
Violin plots comparing the Idu/CldU ratios for relevant plots to the control plot (beige box and lines) from **a** Figure 4b, **b** Figure 4c, **c** Figure 4d, **d** Figure 4e, **e** Figure 4f, **f** Figure 5a, **g** Figure 5b, **h** Figure 5d, and **I** Figure 6a to the WT values with mean (solid line) and quartiles (dashed lines) indicated.

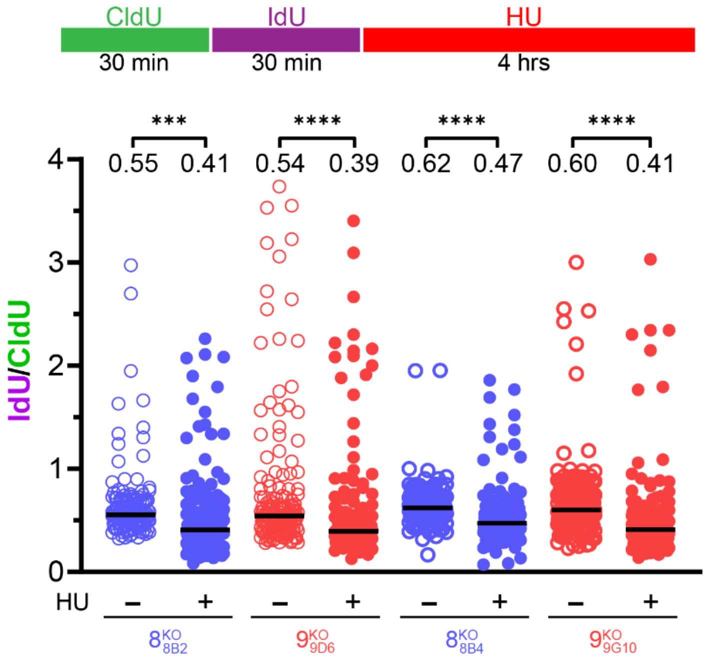

Supplemental Figure 10. Replication forks are consistently unstable in second MCM8 or MCM9 knockout clones.
8^{KO} (8B2 or 8B4 clones, blue) or 9^{KO} (9D6 or 9G10 clones, red) were labeled with CldU followed by IdU for the indicated time intervals followed by 2 mM HU for 4 hours. DNA was spread by gravity to measure replication fork stability and representative fibers are shown. IdU and CldU lengths for were measured using ImageJ and the corresponding median ratios reported at the top of the plots and by a black line embedded in the data. Open circles (○) represent nontreated (NT) conditions, while solid closed circles (●) represent HU treated cells. A Mann-Whitney two-sided U test was used to calculate *P* values (***P< 0.001, ****P< 0.0001).

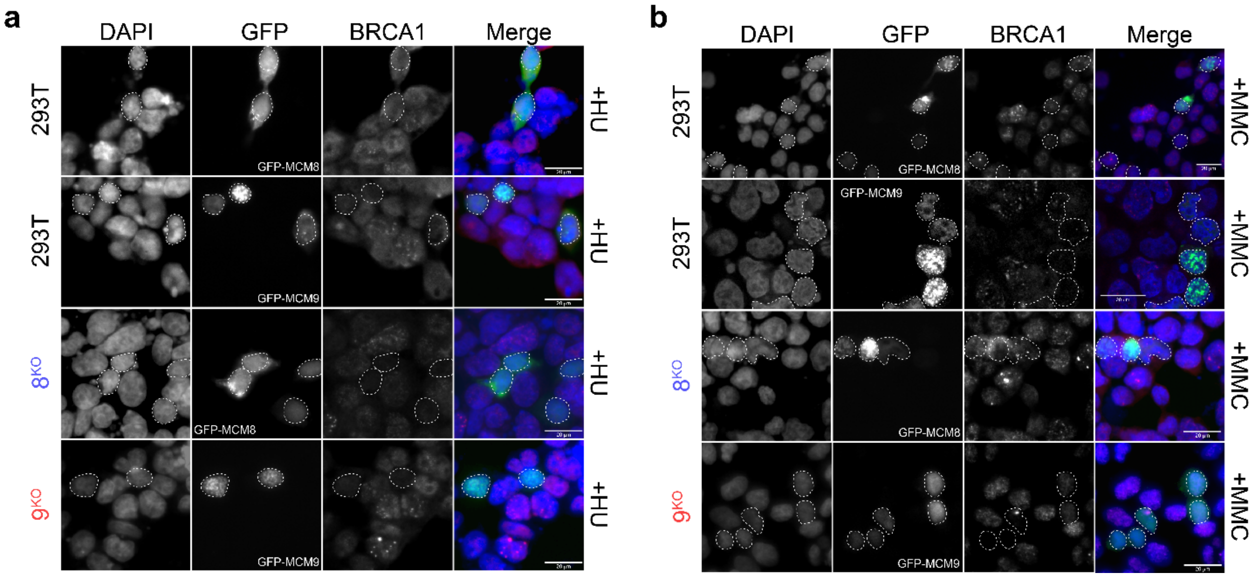

Supplemental Figure 11. Broader views showing MCM8/9 suppression of BRCA1 staining and foci.
293T WT, 8^{KO}, or 9^{KO} cells were transfected with GFP-MCM8 or GFP-MCM9 and treated with 2 mM HU for 4 hours or **b** 3 µM MMC for 6 hours (*n* = 2 independent experiments). Dashed white outlines of nuclei indicate cells successfully transfected and show a decrease in BRCA1 staining and foci. Quantification of prominent BRCA1 foci was done in Figure 6c-d. Scale bars represent 20 µm.
