# Supplementary material for: A multi-functional role for the MCM8/9 helicase complex in maintaining fork integrity during replication stress
Source: Nat Commun. 2022 Aug 30;13:5090. doi: 10.1038/s41467-022-32583-8 (PMC9427862; doi:10.1038/s41467-022-32583-8)
Supplement: Supplementary file 3 — Reporting Summary [file 41467_2022_32583_MOESM3_ESM.pdf]

nature portfolio

Corresponding author(s): Michael Trakselis

Last updated by author(s): Jul 21, 2022

Reporting Summary

Nature Portfolio wishes to improve the reproducibility of the work that we publish. This form provides structure for consistency and transparency in reporting. For further information on Nature Portfolio policies, see our Editorial Policies and the Editorial Policy Checklist.

Statistics

For all statistical analyses, confirm that the following items are present in the figure legend, table legend, main text, or Methods section.

n/a	Confirmed	
☐	☒	The exact sample size (*n*) for each experimental group/condition, given as a discrete number and unit of measurement
☐	☒	A statement on whether measurements were taken from distinct samples or whether the same sample was measured repeatedly
☐	☒	The statistical test(s) used AND whether they are one- or two-sided<br>*Only common tests should be described solely by name; describe more complex techniques in the Methods section.*
☐	☒	A description of all covariates tested
☐	☒	A description of any assumptions or corrections, such as tests of normality and adjustment for multiple comparisons
☐	☒	A full description of the statistical parameters including central tendency (e.g. means) or other basic estimates (e.g. regression coefficient) AND variation (e.g. standard deviation) or associated estimates of uncertainty (e.g. confidence intervals)
☐	☒	For null hypothesis testing, the test statistic (e.g. F, t, r) with confidence intervals, effect sizes, degrees of freedom and P value noted<br>*Give P values as exact values whenever suitable.*
☒	☐	For Bayesian analysis, information on the choice of priors and Markov chain Monte Carlo settings
☒	☐	For hierarchical and complex designs, identification of the appropriate level for tests and full reporting of outcomes
☒	☐	Estimates of effect sizes (e.g. Cohen's *d*, Pearson's *r*), indicating how they were calculated

Our web collection on statistics for biologists contains articles on many of the points above.

Software and code

Policy information about availability of computer code

Data collection	Cell Sens Dimension 2 software (Olympus), FACSVerse (BD Biosciences)
Data analysis	ImageJ 1.52a Software64, Fluoview (v.4.2b), FlowJo v10 (BD Bioscience), GraphPad Prism (v.9.4)

For manuscripts utilizing custom algorithms or software that are central to the research but not yet described in published literature, software must be made available to editors and reviewers. We strongly encourage code deposition in a community repository (e.g. GitHub). See the Nature Portfolio guidelines for submitting code & software for further information.

Data

Policy information about availability of data

All manuscripts must include a data availability statement. This statement should provide the following information, where applicable:

- Accession codes, unique identifiers, or web links for publicly available datasets
- A description of any restrictions on data availability
- For clinical datasets or third party data, please ensure that the statement adheres to our policy

Source data are provided with this paper. Raw data and images are also available upon reasonable request. No new codes were generated.

Field-specific reporting

Please select the one below that is the best fit for your research. If you are not sure, read the appropriate sections before making your selection.

☒ Life sciences ☐ Behavioural & social sciences ☐ Ecological, evolutionary & environmental sciences

For a reference copy of the document with all sections, see nature.com/documents/nr-reporting-summary-flat.pdf

Life sciences study design

All studies must disclose on these points even when the disclosure is negative.

Sample size	Sample size for all experiments include: DNA fibers (>100 fibers from 2 or more independent experiments), comets (>50 comets from 2 or more independent experiments), nuclear foci (>150 foci from 2 or more independent experiments) were chosen to obtain statistical power in conformity with accepted standard sample sizes in the field from previous publications using these approaches:<br>Krishnamoorthy et al, Mol Cell, DOI: 10.1016/j.molcel.2021.05.014<br>Schlacher et al, Cell, DOI: 10.1016/j.cell.2011.03.041<br>Taglialatela et al, Mol. Cell, DOI: 10.1016/j.molcel.2017.09.036
Data exclusions	No data was excluded
Replication	The number of biological replicates are indicated and the representative data is shown in the figures.
Randomization	Asynchronous cell populations were used in the DNA fiber, siRNA, and fluorescence/immunoflourescence cellular imaging providing inherent randomized allocation of cell cycle populations, hence further randomization is not necessary for our approaches. DNA fibers images and measurements will only be obtained from replicating cells in S-phase.
Blinding	DNA fiber collections and quantifications were performed by three separate investigators and results compared between them showed no significant differences. Fiber length measurements were blinded between investigators after acquisition. Identical parameters between samples were used for automatic foci counting, hence further blinding during analysis was not necessary. Western blots were performed in duplicate for qualitative analysis and were therefore unblinded. FACS analysis and histograms was provided automatically by the software and were therefore not blinded to the investigators.

Reporting for specific materials, systems and methods

We require information from authors about some types of materials, experimental systems and methods used in many studies. Here, indicate whether each material, system or method listed is relevant to your study. If you are not sure if a list item applies to your research, read the appropriate section before selecting a response.

Materials & experimental systems

n/a	Involved in the study	
☐	☒	Antibodies
☐	☒	Eukaryotic cell lines
☒	☐	Palaeontology and archaeology
☒	☐	Animals and other organisms
☒	☐	Human research participants
☒	☐	Clinical data
☒	☐	Dual use research of concern

Methods

n/a	Involved in the study	
☒	☐	ChIP-seq
☐	☒	Flow cytometry
☒	☐	MRI-based neuroimaging

Antibodies

Antibodies used	Primary Antibodies: MCM8 (Proteintech, 16451-1-AP), MCM9 (ThermoFisher, PA5-113440), SMARCAL1 (Bethyl Laboratories, A301-616A), HLTF (Bethyl Laboratories, A300-640A), BRCA1 (Santa Cruz, sc-6954), MRE11 (Proteintech, 10744-1-AP), DNA2 (Invitrogen, PA5-68167), EXO1 (Bethyl Laboratories, A302-640A), MUS81 (Abcam, ab14387), gamma-H2A.Xp (Abcam, ab26350), GAPDH (Pierce, MA5-15738), Lamin B1 (Proteintech, 12987-1-AP), beta-actin (Abcam, ab82227), mouse anti-BrdU (BD Bioscience, BD-347580) and rat anti-BrdU (Abcam, ab6325).<br>Secondary antibodies: goat anti-rabbit HRP (Novex, A16096), goat anti-mouse HRP (Novex, A16072), anti-mouse-Cy3-conjugated (Abcam, 97035), anti-rat 488-conjugated (Abcam, 150157), anti-mouse Alexa647 (ThermoFisher, A-21235).
Validation	All antibodies used in this study are commercially available (see catalog numbers above) and show the band of the expected size. siRNA knockdown of associated genes show corresponding decreases in protein intensities by western blot in several Figures throughout the manuscript. Validation and verification experiments of the specificity for MCM8, SMARCAL1, HLTF, MRE11, DNA2, EXO1, Lamin B1, and beta-actin in western-blotting are displayed on the corresponding manufacturer's website.

Eukaryotic cell lines

Policy information about cell lines

Cell line source(s)	Parental HEK293T (ATCC CRL-3216) and MCM8 and MCM9 knockout cell lines were previously described in: McKinzey 2020, JBC, DOI: 10.1016/j.jbc.2021.100355. Two separate MCM8 and MCM9 knockout clones (each) were validated in this manuscript.
Authentication	DNA sequencing analysis of the MCM8 and MCM9 gene knockout regions were previously validated in: McKinzey 2020, JBC, DOI: 10.1016/j.jbc.2021.100355. No further authenticaion of the cell lines were done for this manuscript.
Mycoplasma contamination	Cell lines have tested negative for mycoplasma.
Commonly misidentified lines (See ICLAC register)	No commonly misidentified lines were used in this study.

Flow Cytometry

Plots

Confirm that:

- [x] The axis labels state the marker and fluorochrome used (e.g. CD4-FITC).
- [x] The axis scales are clearly visible. Include numbers along axes only for bottom left plot of group (a 'group' is an analysis of identical markers).
- [x] All plots are contour plots with outliers or pseudocolor plots.
- [x] A numerical value for number of cells or percentage (with statistics) is provided.

Methodology

Sample preparation	293T, 8KO, or 9KO cells were synchronized at the beginning of S-phase using a double thymidine block as described in Materials and Methods.
Instrument	FACSVerse (BD Biosciences)
Software	FlowJo (BD Bioscience, v10)
Cell population abundance	No sorting was performed.
Gating strategy	Cell cycle determination was analyzed using forward scatter (FSc) and side scatter (SSc), selecting for unaggregated live cells.

- [x] Tick this box to confirm that a figure exemplifying the gating strategy is provided in the Supplementary Information.
